# Missing A1 Unmasked During Surgery for Ruptured Anterior Communicating Artery (ACoA) Aneurysm: Navigating the Unexpected

**DOI:** 10.7759/cureus.96372

**Published:** 2025-11-08

**Authors:** A Sathia Prabhu, Karthik Nandam, Praveen Ravichandran

**Affiliations:** 1 Neurosurgery, Jawaharlal Institute of Postgraduate Medical Education and Research, Puducherry, IND; 2 Neurosurgery, SIMS Hospital, Chennai, IND

**Keywords:** aca vascular anomaly, acom aneurysm with vascular anomaly, carotid-aca anastomosis, infraoptic aca, missing a1

## Abstract

The infraoptic anterior cerebral artery (ACA) is a rare vascular anomaly that is often regarded as merely an anatomical curiosity. We report the case of a 54-year-old hypertensive man who presented with subarachnoid hemorrhage (World Federation of Neurological Surgeons (WFNS) Grade 4) due to rupture of a bilobed anterior communicating artery (ACoA) aneurysm. CT angiography confirmed the aneurysm, and the patient underwent right pterional craniotomy and clipping. Intraoperatively, the right A1 segment was absent in its usual supraoptic location and was instead found coursing beneath the optic nerve, consistent with a Wong type II infraoptic ACA. The aneurysm was successfully clipped with preservation of perforators, and postoperative angiography confirmed complete occlusion. This case highlights the importance of recognizing an infraoptic A1 variant in patients with a “missing” A1 segment on imaging or during ACoA aneurysm surgery, as failure to identify this anomaly may compromise surgical safety and completeness.

## Introduction

The anterior cerebral artery (ACA) typically arises from the bifurcation of the internal carotid artery (ICA) at the level of the anterior perforated substance [1]. From its origin, the A1 segment courses anteromedially above the optic nerve and optic chiasm, lying within the carotid cistern before joining its contralateral counterpart via the anterior communicating artery (ACoA) at the base of the lamina terminalis [2,3]. The A1-A2 junction and ACoA complex form an important microsurgical corridor, bordered laterally by the optic nerves, superiorly by the gyrus rectus, and inferiorly by the chiasmatic cistern, making precise anatomical orientation essential during aneurysm surgery [3,4].

In rare instances, the ACA originates lower than usual, taking a course beneath the optic nerve, a variant known as the infraoptic ACA. Fewer than 60 such cases have been reported, although this anomaly is likely underrecognized due to its subtle imaging appearance and infrequent clinical detection [5,6].

While traditionally regarded as an anatomical curiosity, the infraoptic ACA has critical clinical relevance when the A1 segment is absent in its expected suprasellar position. In such cases, particularly with an ACoA aneurysm, failure to recognize an infraoptic A1 may lead to misinterpretation of vascular anatomy and compromise surgical or endovascular planning. This variant is also occasionally associated with contralateral A1 hypoplasia, asymmetric hemodynamics, and a predilection for aneurysm formation at the ACoA complex [7,8].

We present the case of a ruptured ACoA aneurysm in which both A1 segments followed an infraoptic course. This case underscores the importance of recognizing this variant, not merely as an embryological curiosity, but as a critical diagnostic and surgical consideration when the A1 segment is not clearly visualized on preoperative imaging.

## Case presentation

The patient presented in July 2023 with an acute-onset, severe holocranial headache that began that morning. The headache progressively worsened and was associated with two episodes of projectile vomiting and a transient loss of consciousness. On arrival, he was drowsy but arousable, with a Glasgow Coma Score (GCS) of E3V3M5, corresponding to a WFNS Grade IV. He had a recent diagnosis of hypertension and was on medication. The pupils were bilaterally equal and reactive to light, and no focal neurological deficits or cranial nerve palsies were noted at admission.

**Figure 1:**
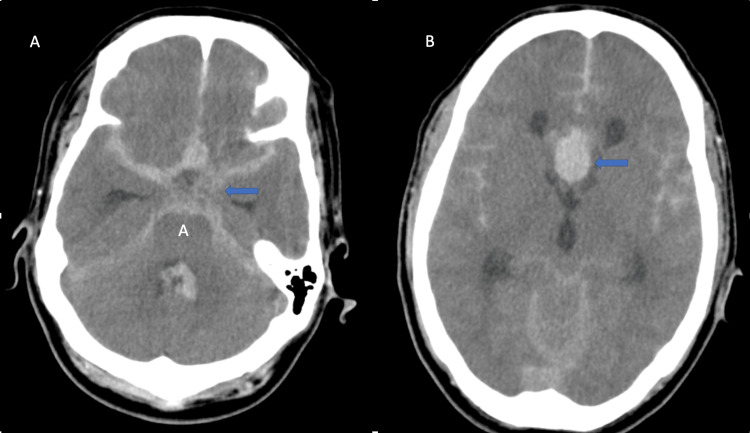
Plain CT of the brain (axial sections) showing diffuse, thick subarachnoid hemorrhage involving the perimesencephalic and suprasellar cisterns (blue arrow in A) and bilateral Sylvian cisterns, with the densest collection in the interhemispheric fissure (blue arrow in B). Associated intraventricular hemorrhage and ventriculomegaly are noted, corresponding to a modified Fisher Grade 4

**Figure 2:**
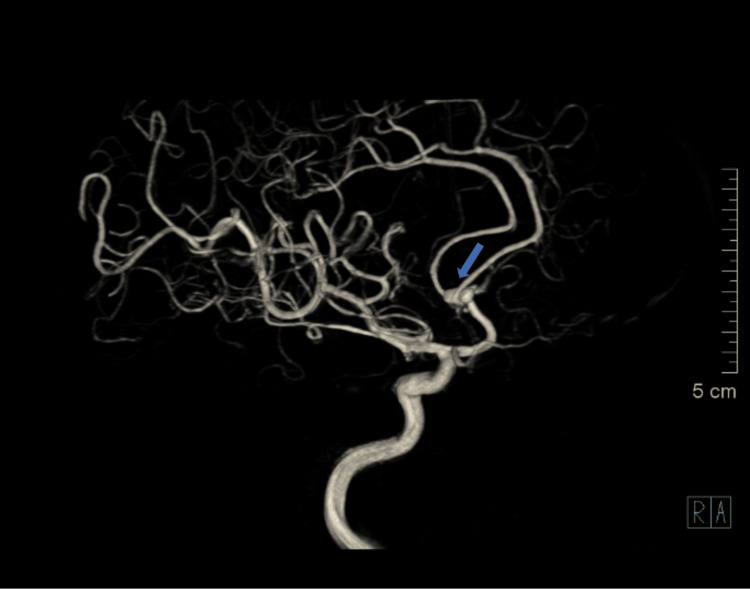
Digital subtraction angiography (DSA) following right internal carotid artery (ICA) injection demonstrating a bilobed saccular aneurysm (blue arrow) at the anterior communicating artery (ACoA)

A non-contrast CT brain revealed diffuse, thick subarachnoid hemorrhage (SAH) involving the basal cisterns with intraventricular extension and mild ventriculomegaly, consistent with a Modified Fisher Grade 4 (Figure 1). Digital subtraction angiography (DSA) demonstrated a bilobed aneurysm of the ACoA (Figure 2). The left A1 segment was hypoplastic.

**Figure 3:**
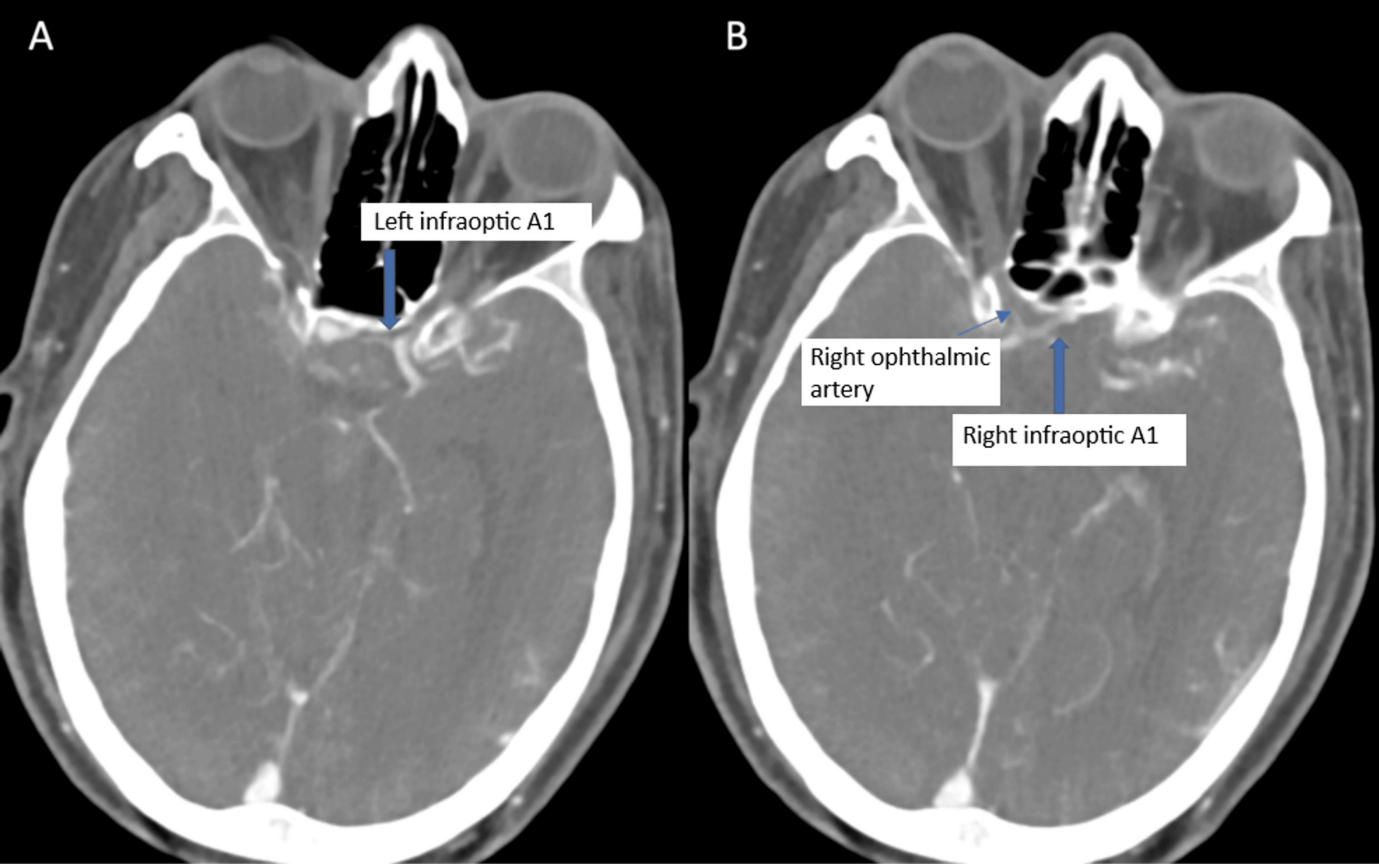
Axial CT cerebral angiography demonstrating bilateral infraoptic courses of the anterior cerebral arteries (ACAs) (A) Left infraoptic A1 segment (blue arrow). (B) Right infraoptic A1 segment (blue arrow) and right ophthalmic artery (blue arrowhead). The A1 segments arise from the internal carotid arteries (ICA) and course beneath the optic nerves before turning medially toward the anterior communicating artery (ACoA) complex.

The patient underwent a standard right pterional craniotomy using a subfrontal approach for aneurysm clipping. Intraoperatively, the right A1 segment was not visualized in its expected suprasellar location, despite clear identification of the ICA, optic nerves, and interoptic space (Video 1). Careful dissection through the carotid-optic and interoptic spaces, where the A1 segment typically courses above the optic nerve, failed to reveal it. Intraoperative review of the CT cerebral angiogram (Figure 3) demonstrated that both ICAs bifurcated at an unusually low level, near the origin of the ophthalmic artery.

**Video 1:** Intraoperative video showing clipping of the ACoA aneurysm with bilateral infraoptic ACA 0:04 We present the case of a missing A1 that was unmasked during surgery for a ruptured anterior communicating artery aneurysm. 0:11 The right ICA injection during DSA showed a bilobed saccular aneurysm at the anterior communicating artery projecting medially. The left A1 segment is hypoplastic. 0:23 We did a right pterional craniotomy and drilled the sphenoid ridge. Sylvian dissection was done. We exposed the right optic nerve. We continued dissection in the right optico-carotid cistern in anticipation of the usual suprasellar course of A1, which courses above the optic nerve. But it was not found. 0:43 Intraoperative CT cerebral angiogram review showed that the bilateral ICA bifurcation was rather lower than the usual location, at the level of the ophthalmic artery. Bilateral A1 segments were seen to course anteriorly toward the tuberculum sella and then posterosuperiorly to join at the anterior communicating artery. Correlating with intraoperative findings, we found out that these were bilateral infraoptic A1, rare anatomical variants. 1:07 We continued dissection in the interoptic space to visualize the bilateral infraoptic A1 segments. The dominant right A1 was found superior to the left hypoplastic A1. We visualized the left optic nerve. Once the bilateral A1 segments were clearly defined, we proceeded with the resection of the ipsilateral gyrus rectus. 2:11 After further dissection, we were able to define the junction of the A1 and A2 with the neck of the aneurysm. 2:21 We placed the temporary clips on the bilateral A1 segments and proceeded with further dissection of the dome. We secured the aneurysm with two permanent clips. We then readjusted the clips to ensure complete clipping of the aneurysm.  Post-clipping fluorescent imaging confirmed complete aneurysm occlusion, parent vessel, and perforator vessel patency. 2:51 Postoperative DSA confirmed no residual aneurysmal neck and no vasospasm. His GCS improved to E4V4M6, and he had no deficits.

We observed that the bilateral ICA bifurcations were positioned lower than usual, at the level of the ophthalmic arteries. Both A1 segments coursed anteriorly toward the tuberculum sella and then turned posterosuperiorly to join at the ACoA. Correlating with intraoperative findings, these were identified as bilateral infraoptic A1 segments, a rare anatomical variant [2,3,5]. The ACoA, right A1 and A2 segments, and the recurrent arteries of Heubner were identified, and the bilobed aneurysm was traced from these vessels. The aneurysm was successfully clipped using two curved 7 mm titanium clips, with careful preservation of the perforators (Video 1).

**Figure 4:**
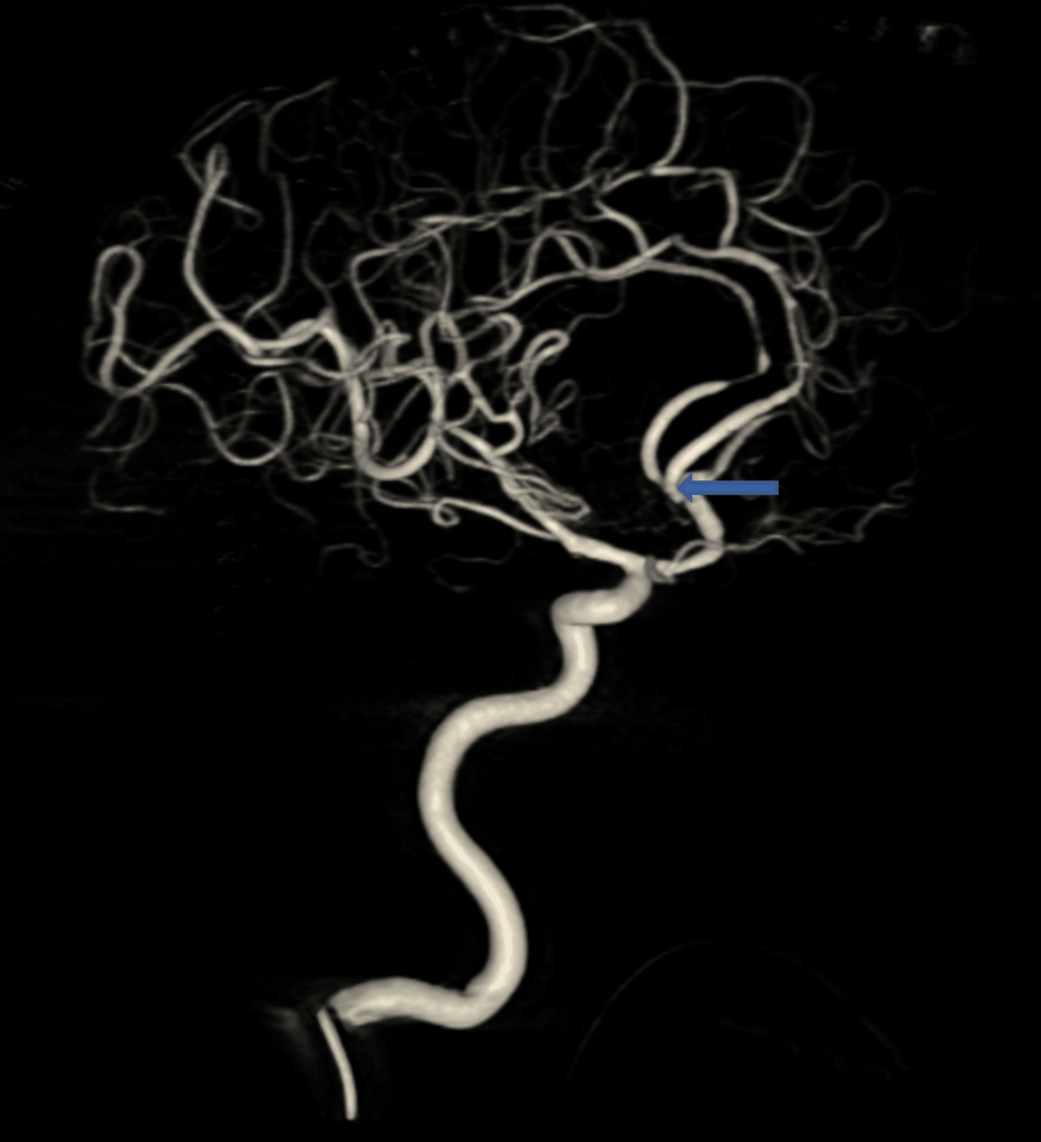
Postoperative digital subtraction angiography (DSA) showing complete occlusion of the aneurysm. The blue arrow indicates the site of the previously visualized anterior communicating artery (ACoA) aneurysm, now absent following successful clipping

Postoperative DSA confirmed complete aneurysm occlusion (Figure 4). Due to persistently high external ventricular drain output, the patient underwent conversion to a right Frazier ventriculoperitoneal shunt. His GCS improved to E4V4M6 postoperatively, with no neurological deficits. At three-month outpatient follow-up, his GCS was E4V5M6, and he remained neurologically intact.

## Discussion

ACoA aneurysms remain the most common among ruptured aneurysms treated with clipping or coiling [9]. This emphasizes the need for a clear and precise understanding of vascular anatomy before any intervention, particularly surgical clipping. Anomalous variants of the ACA can pose significant intraoperative challenges if they are not recognized preoperatively on angiography. The infraoptic ACA is a rare vascular anomaly that usually arises from the intradural segment of the ICA, near the origin of the ophthalmic artery. It has also been referred to as the carotid-ACA anastomosis. The vessel courses beneath the ipsilateral optic nerve, then passes between the optic nerves to reach the prechiasmatic cistern, eventually joining the ACA-ACoA complex. Rather than being viewed merely as an anatomical curiosity, this variant should be suspected whenever the A1 segment is not visualized in its usual supraoptic location. 

The infraoptic ACA and its variations have been described by 55 others previously. It was first reported by Robinson in 1959 [10]. This anomaly is extremely rare, with an estimated prevalence of about 0.086% on MR angiography, showing a right-sided predominance [6]. Several theories have been proposed regarding its embryological origin [8,11]. It is thought to result from abnormal persistence or regression of embryonic vessels, leading to the ACA originating at or near the level of the ophthalmic artery, and in some cases, even from an extradural segment of the ICA. The vessel then courses beneath the optic nerve(s) to reach the interhemispheric fissure [5,12]. This anomaly is frequently associated with a low bifurcation of the ICA and may occur unilaterally or bilaterally [5,13]. Another plausible explanation for its development is defective formation of the rostrolateral arterial ring around the optic nerves and chiasm, which normally gives rise to the ophthalmic and anterior cerebral arteries bilaterally [2]. In a review of 47 similar cases, this variant consistently demonstrated three features: the infraoptic ACA coursed beneath the optic nerve, arose from the ICA at the level of the ophthalmic artery, and the anastomosis between the ACoA and ACA supplied the vascular territory of a normal ACA [7].

Wong et al. proposed a classification system for infraoptic ACA anomalies, highlighting the anatomical diversity that may be encountered in cerebrovascular surgery [14]. In Type I, the infraoptic A1 functions as a collateral vessel in addition to the normal ACA-ACoA configuration. Type II involves a low bifurcation of the ICA, where the infraoptic A1 replaces the standard A1 segment. Type III is similar to Type II but is characterized by the absence of the contralateral A1. Finally, Type IV describes an accessory ACA that arises independently, without merging into the typical ACA-ACoA network. Our case most closely corresponded to Wong Type II.

A review of the literature shows that a significant proportion of reported infraoptic ACA cases are associated with ruptured or unruptured aneurysms, most commonly at the ACoA (Table 1). This vascular anomaly appears to predispose patients to aneurysm formation, particularly within the ACA-ACoA complex. Approximately 59% of reported cases were associated with cerebral aneurysms, and 44%-50% of patients with infraoptic ACAs developed anterior communicating artery aneurysms [2,3,15-19]. In addition, this anomaly may coexist with other vascular malformations, such as agenesis of the contralateral ICA or aortic coarctation [13,20].

**Table 1:** Summary of reported cases and literature on infraoptic anterior cerebral artery (ACA)

Author (year)	Study type	Anomaly/topic	No. of cases	Salient findings
Kochar et al. (2021) [5]	Case series and literature review	Infraoptic ACA/carotid-ACA anastomosis	3 cases	Discussed embryological mechanisms and clinical significance; emphasized association with aneurysms
Uchino et al. (2012) [6]	Observational study	Carotid-ACA anastomosis detected on MR angiography	1,500 MR angiograms; prevalence 0.2%	Non-invasive radiological identification and prevalence study
Vogel et al. (1952) [1]	Embryological and pathological study	Anomalies of major cerebral arteries with congenital brain malformations	Histopathological correlation	Proposed embryological mechanisms leading to infraoptic ACA formation
Kang et al. (2012) [20]	Case report	Ruptured aneurysm at infraoptic azygous ACA with contralateral ICA agenesis	1 case	Y-stent-assisted coil embolization successfully performed
Tanaka et al. (2021) [3]	Case report	Ruptured ACoA aneurysm with infraoptic ACA	1 case	Microsurgical clipping; reviewed 15 prior cases
Turkoglu et al. (2011) [7]	Case report	ACoA aneurysm with infraoptic ACA and persistent trigeminal artery variant	1 case	Microsurgical clipping; embryologic analysis of dual anomaly
Chakraborty et al. (2006) [15]	Case report	Bilateral infraoptic origin of ACA	1 case	Incidental discovery; implications for endovascular procedures
Pang et al. (2023) [12]	Case report	Unusual infraoptic ACA variant	1 case	Anatomical variation documented radiologically
Ji et al. (2009) [13]	Case report	Infraoptic ACA with MCA aneurysm and aortic coarctation	1 case	Described on angiography, a rare combination
Wong et al. (2008) [2]	Review + 2 case reports	Infraoptic ACA classification and literature review	2 cases	Proposed classification system based on embryologic origin
Bollar et al. (1988) [8]	Case report	Anomalous origin of ACA associated with aneurysm	1 case	Embryological discussion; angiographic documentation
Brismar et al. (1977) [11]	Case report	Anomalous ACA origin	1 case	Embryologic considerations; imaging-based diagnosis
Bosma (1977) [21]	Case report	Infraoptic ACA with low ICA bifurcation	1 case	Anatomical and radiological correlation
Lehmann et al. (1980) [18]	Case report	Infraoptic pathway of ACA	1 case	Bilateral variant; discussed rare configuration of the circle of Willis
Fujimoto et al. (1983) [16]	Case report	Anomalous ICA branch supplying ACA circulation	1 case	Radiological and surgical correlation
Isherwood and Dutton (1969) [15]	Case report	Unusual ACA anomaly	1 case	Angiographically detected; discussed the embryological background
Kessler (1979) [19]	Case report	Unusual ACA anomaly	1 case	Single variant observed radiologically
Orz and AlYamany (2015) [9]	Retrospective clinical study	Impact of size/location on aneurysm rupture	Clinical data analysis	Referenced for aneurysm rupture risk context

This anomaly can cause visual deficits in unruptured cases due to atherosclerotic changes [21]. Intraoperatively, defining the aneurysm and its relationship to the ACA branches is challenging because the proximal ACA lies hidden beneath the optic nerve, leading to spatial disorientation. Surgical management is further complicated by the difficulty in obtaining proximal control through a narrow corridor and the risk of injury to the optic apparatus or the ophthalmic artery. In our case, the variant anatomy was not evident preoperatively and was only recognized when the aneurysm’s location proved difficult to trace. Failure to identify this anomaly beforehand can result in unexpected dissection and potential injury to the optic pathways or basal frontal lobes. Furthermore, the vertical midline segment of the proximal ACA often forms a superiorly directed aneurysm positioned relatively high [20], requiring meticulous dissection around the optic nerve and surrounding structures to safely expose and clip the aneurysm neck. 

## Conclusions

While the infraoptic course of the ACA is a rare developmental anomaly, its importance extends beyond being an anatomical curiosity. In surgical practice, the infraoptic ACA should be actively considered when the A1 segment is not visualized in its typical supraoptic location. Failure to recognize this variant can misidentify vascular anatomy and compromise safe aneurysm dissection and clipping. This case highlights the critical need for high-resolution preoperative vascular imaging and a vigilant intraoperative search for variant anatomy, particularly in ACoA aneurysms. Early recognition of infraoptic ACA can significantly influence surgical planning and reduce the risk of complications.
